# Interaction of Terminal Oxidases with Amphipathic Molecules

**DOI:** 10.3390/ijms24076428

**Published:** 2023-03-29

**Authors:** Natalia V. Azarkina, Vitaliy B. Borisov, Ilya P. Oleynikov, Roman V. Sudakov, Tatiana V. Vygodina

**Affiliations:** Belozersky Institute of Physico-Chemical Biology, Lomonosov Moscow State University, Leninskie Gory 1, Bld. 40, 119992 Moscow, Russia

**Keywords:** molecular bioenergetics, terminal oxidases, cytochrome oxidase, bile acid-binding site, amphipathic ligands, tight bound lipids, detergents, regulation

## Abstract

The review focuses on recent advances regarding the effects of natural and artificial amphipathic compounds on terminal oxidases. Terminal oxidases are fascinating biomolecular devices which couple the oxidation of respiratory substrates with generation of a proton motive force used by the cell for ATP production and other needs. The role of endogenous lipids in the enzyme structure and function is highlighted. The main regularities of the interaction between the most popular detergents and terminal oxidases of various types are described. A hypothesis about the physiological regulation of mitochondrial-type enzymes by lipid-soluble ligands is considered.

## 1. Introduction

Terminal oxidase is a key enzyme in the respiratory chain of mitochondria and aerobic bacteria. It is located in the coupling membrane and catalyzes the transfer of electrons from the respiratory substrate (cytochrome *c* or membrane quinol) to oxygen. This reaction is coupled to the generation of a proton motive force (∆μH^+^) across the membrane. The four-electron oxygen reduction proceeds in the binuclear catalytic center. Within the numerous members of the heme–copper superfamily, the binuclear center comprises a high-spin heme and a copper ion, Cu_B_, whereas among the *bd*-type enzymes, it consists of two high-spin hemes [1].

During the catalytic cycle, protons are transported through intra-protein proton channels. The number and structure of the proton channels underlie the classification of the heme–copper oxidases into types A (further subdivided to A1 and A2), B, and C [2,3,4,5,6]. High-resolution crystal structures are currently available for members of each type. All heme–copper oxidases possess proton-pumping activity, but its mechanism differs somewhat between the A, B, and C types [6,7,8,9,10]. The type A1 enzymes, including the cytochrome *c* oxidase (CcO) of mitochondria, are the most studied [11,12,13]. Cytochrome *c* oxidases from *Paracoccus denitrificans* [14,15] and *Rhodobacter sphaeroides* [16,17], as well as the quinol oxidase *bo*_3_ from *Escherichia coli* [18,19], are the best studied type A bacterial enzymes. Type B terminal oxidases are found in Eubacteria and Archea. A classic example is the cytochrome *c* oxidase *ba*_3_ from *Thermus thermophilus* [20,21,22]. Type C, represented by the cytochrome *c* oxidase *cbb_3,_* is phylogenetically, and structurally, the most distant from type A [10]. They are common in microaerophilic Proteobacteria [23,24].

Terminal *bd*-type quinol oxidases (syn. cytochrome *bd*) are found in a wide range of Eubacteria and Archea living under low oxygen conditions [25,26]. They are structurally and evolutionarily different from heme–copper oxidases, and constitute their own superfamily divided into S and L groups, which differ in the size of the quinol-binding domain [26]. The 3D structures of both S and L representatives have been published [27,28]. In contrast to heme–copper oxidases, *bd*-oxidases do not pump protons, but still generate ∆μH^+^ due to the spatial separation of the reactions proceeding with the consumption and release of protons [29]. They are highly resistant to the most common inhibitors of heme–copper enzymes [30,31].

Mitochondrial CcO consists of three major subunits encoded by the mitochondrial genome, and ten minor regulatory subunits encoded within the nucleus [32]. The properties of the isolated preparation significantly depend on the preservation of subunits during solubilization and purification. The main catalytic subunit I contains low-spin heme *a* and an oxygen reductive binuclear center, *a*_3_/Cu_B_. Subunit II interacts with the soluble cytochrome *c*, and harbors the “input” redox center, Cu_A_. Bacterial terminal oxidases usually contain two to four subunits. Among the heme–copper enzymes, they are homologous to the main subunits of the mitochondrial CcO.

Well-studied *E. coli* cytochrome *bd*-I consists of four subunits: CydA, CydB, CydX, and CydH (also named CydY) [28,33]. CydA harbors a quinol-binding site and three hemes, *b*_558_, *b*_595_, and *d*. The low-spin heme *b*_558_ directly participates in the oxidation of quinol. The high-spin hemes *b*_595_ and *d* form an oxygen-reducing site.

Terminal oxidases are integral membrane proteins and, as such, have a predominantly hydrophobic surface that is in contact with membrane lipids in vivo. In the isolated preparation, the natural lipid is mostly replaced by detergent molecules. The incorporation of the enzyme into detergent micelles determines its solubility in aqueous media. Besides the solubilized form, membrane proteins are commonly studied in proteoliosomes. In this case, the previously purified enzyme is incorporated into an artificial membrane, the lipid composition of which may differ significantly from the natural one. Catalytically active preparations usually contain a certain number of “tightly bound”, native lipid molecules. Individual phospholipid molecules are visible in the crystal structures of the terminal oxidases. Some of them are assumed to have a direct effect on the conformation and the activity of the enzyme.

Mitochondrial CcO can exist in both monomeric and dimeric forms. Both forms are catalytically active, although they differ in terms of the activity and efficiency of ∆μH^+^ generation. According to the current knowledge, both states are physiological, and the transition between them has a regulatory significance [34]. CcO preparations can convert into a dimeric or monomeric state, depending on the isolation procedure. In particular, the detergent used for solubilization plays a decisive role. It has been shown that CcO can form a catalytically active supercomplex with other enzymes of the respiratory chain [35]. Unlike mitochondrial CcO, its bacterial analogs are known only as monomers [36].

Four pathways regarding the intracellular regulation of CcO activity are known: tissue-specific changes in subunit composition [32], reversible changes in quaternary structure [34,36], covalent modifications (phosphorylation and acetylation) of certain residues [34,37] and reversible interactions of the enzyme with ligands. For a long time, only low-molecular-weight, water-soluble substances remained examples of the latter: adenine nucleotides’ binding on the matrix-facing surface of subunit IV [38], some gas molecules interacting with the binuclear catalytic center as oxygen mimetics [39], and calcium ions that bind at the conserved site of the subunit I, near the outer surface of the membrane [40].

The situation changed about 15 years ago, when the site of interaction with lipid-soluble ligands in the structure of the mitochondrial CcO dimer was described [41]. It is located at the junction of subunits I, II and VIa, and has an affinity for many physiologically active, amphipathic compounds [42,43], including the bile acid anions, due to which it was named the Bile Acid-Binding Site (BABS). It is probable that BABS is characteristic of type A heme–copper oxidases. Interestingly, an alternative binding site for amphipathic ligands has recently been described in the structure of the monomeric CcO from mitochondria [44]. Apparently, in vivo, mitochondrial CcO may be controlled by an activity regulation system that is multispecific, with respect to physiologically significant lipophilic molecules. Among the putative BABS ligands, natural and artificial steroid compounds, thyroid hormones, nucleotides, heme derivatives, etc., and even small cell penetrating peptides, have been described. The interaction of some detergents with BABS is an interesting special case of their influence on the activity of CcO.

Taking into account the above, in this review, we consider the regularities of the effects of various amphipathic compounds on the structure and functioning of terminal oxidases. Substances with such effects belong to the following main groups:

- Lipids.
- Detergents.
- Relatively small amphipathic molecules.

The most common ways of influencing the enzyme seem to be the following: varying the preparation quality (the number of subunits, conformations, etc.); inducing change in the quaternary structure of the enzyme; binding to the regulatory sites that have affinity for endogenous ligands.

## 2. Lipids

Lipid–protein interactions represent a vast area of research. In this review, we do not claim to be complete. We only trace the main patterns and mention some interesting cases.

To obtain a terminal oxidase as a purified preparation, the enzyme, first of all, should be extracted from the native membrane and incorporated into an artificial hydrophobic environment, which imitates the natural lipid environment. In early studies of the mitochondrial CcO, an attempt was often made to purify the protein from native phospholipids as completely as possible. For this purpose, organic solvents [45], treatment with phospholipase [46], as well as precipitation from solution with ammonium sulfate [47], were used. Robinson and Copaldi developed the “soft delipidization” procedure, which, in contrast to the works mentioned above, excluded the stage of converting the enzyme into an insoluble precipitate [48]. It was based on the formation of a water-soluble protein complex with non-denaturing detergent molecules that replace most of the phospholipid molecules.

Pretty soon, it became clear that there exists a population of tightly bound lipid molecules, the removal of which leads to a sharp decrease in the CcO activity. Thus, a purified CcO preparation from the bovine heart was shown to contain the cardiolipin molecules, which were inaccessible to most solvents and phospholipases [46]. Subsequent studies confirmed the presence of stoichiometric amounts of cardiolipin in CcO [48]. The presence of two moles of cardiolipin per monomer of the solubilized enzyme was shown to be necessary for catalytic activity [49]. Later, it was shown that cardiolipin can be almost completely removed from the solubilized CcO by phospholipase A2 [50]. This is accompanied by the loss of the small subunits VIa and VIb, which are involved in the stabilization of the enzyme in the dimeric state [12,51]. Later, this 11-subunit, cardiolipin-lacking CcO monomer turned out to be defective in proton translocation [52]. Some of cardiolipin-binding sites have been predicted by molecular modeling [53]. According to the current knowledge, cardiolipin plays an important role in the physiologically significant reversible transition of eukaryotic CcO from a monomeric to dimeric state [52,54,55,56], as well as in forming supercomplexes with other respiratory enzymes [57].

The presence of cardiolipin in the inner mitochondrial membrane apparently reflects the prokaryotic origin of mitochondria. Indeed, this lipid is typical of bacterial membranes and plays an important role in the formation of supercomplexes in prokaryotic respiratory chains [57]. Besides, cardiolipin has been shown to have a direct, activating effect on the bacterial quinol oxidases *bd* and *bo*_3_, solubilized in dodecyl maltoside [58]. The addition of 10 μM cardiolipin results in a two-fold activation of the oxygen consumption by the enzyme, as well as in two- to three-fold decrease in the apparent *K*_M_ for quinol (both ubi- and menaquinol). Cardiolipin affects the cytochrome *bd* from Gram-negative (*E. coli*), as well as Gram-positive (*Geobacillus thermodenitrificans* and *Corynebacterium glutamicum*), bacteria. The activation mechanism remains unknown. Given the decrease in *K*_M_, it can be assumed that cardiolipin promotes a more efficient interaction with the reducing substrate, membranous quinol. It is known, for example, that cardiolipin binding ensures the correct fit of quinol within the Q_D_ site of the nitrate reductase complex [59]. Further, in the presence of cardiolipin, the enzyme in detergent micelles can be more favorably oriented [60]. The role of cardiolipin may also consist of stabilizing the structure of the enzyme. It probably fills cavities and clefts on the enzyme’s surface [55], and binds at the interface between subunits, as found for formate dehydrogenase-N [61].

According to [48], the treatment of CcO from the bovine heart with either ionic (bile acid salts) or non-ionic (Triton X-100, Tween) detergents allows one to remove most of the native phospholipids; however, 6–10 molecules of the latter per monomer remain bound to the enzyme. It was shown, therein, that CcO forms dimers in Tween and Triton X-100 solutions, while cholate and deoxycholate promote a monomeric state (later, this statement was reconsidered; see below). The authors attributed the observed difference to the delipidization of the preparation, which, as they found, was more complete in the presence of bile acid salts. However, inhibition by (deoxy)cholate could not be explained by this alone, since it was also observed in a preparation containing a significant admixture of native phospholipids.

At the moment, the need for tightly bound lipids in the composition of physiologically active heme–copper oxidases is beyond doubt. In the 1.8 Å crystal structure of the 13 subunit CcO from bovine heart mitochondria (PDB 2DYR), 13 lipid molecules were identified: two cardiolipin molecules, one phosphatidyl choline, three phosphatidyl ethanolamines, four phosphatidyl glycerols, and three triacylglycerols [62]. Tightly bound lipids are also found in bacterial heme–copper oxidases. Thus, in the crystal structure of the four-subunit CcO from *R. sphaeroides* (PDB entry 1M56), six phosphatidyl ethanolamines are visible [17], and in the crystal structure of the four-subunit CcO from *P. denitrificans* (PDB entry 1QLE), two phosphatidyl cholines are observed [63]. The typical binding sites for these lipid molecules are the gaps between the transmembrane α-helices and the grooves at the subunit contacts. Often, in the crystal structures of CcO, tightly bound molecules of the detergent used in crystallization are visible. In this case, the detergent is considered to be a substitute for endogenous lipids. A comparison of crystal structures shows that positions of detergent alkyl chains and endogenous lipid fatty acids are almost exactly the same in both mitochondrial and bacterial CcOs. This allowed the authors to conclude that type A heme–copper enzymes contain conserved lipid-binding sites [64]. This conclusion is confirmed by a comparison of primary sequences encoding transmembrane regions in various oxidases which was carried out in the same work. It reveals conserved sites, corresponding to the contacts of the protein with fatty acids of endogenous lipids and alkyl chains of detergents. These sites coincide well in various members of the type A heme–copper oxidases.

**Figure 1:**
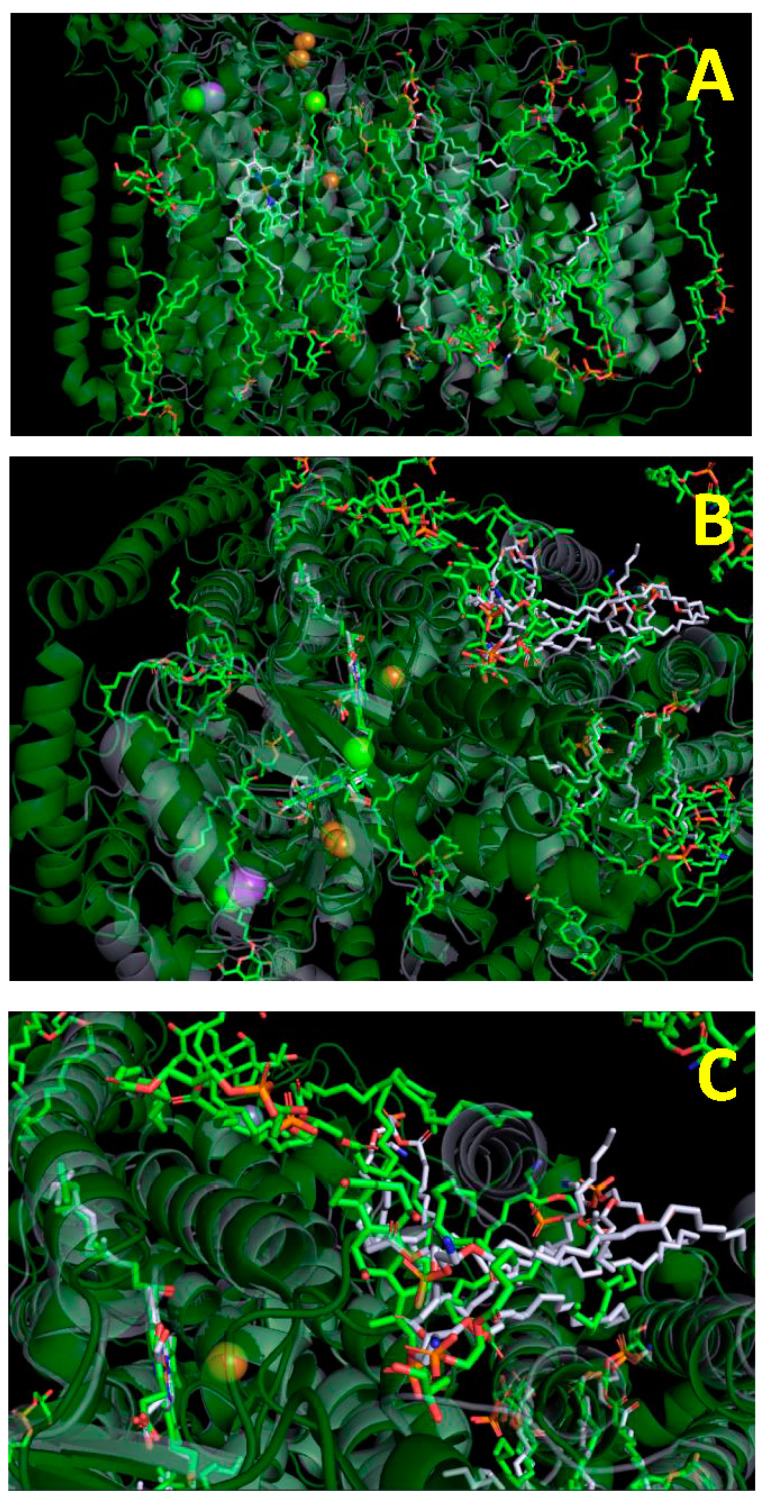
Structure of the type A heme–copper terminal oxidases with tightly bound lipids. 3D structures of dimeric cytochrome *c* oxidase (CcO) from bovine heart mitochondria (PBD entry 3ASO; green) and CcO from *Rhodobacter sphaeroides* (PBD entry 1M56; grey) are superimposed. Lipids are shown by sticks in the corresponding colors, with oxygen (red), nitrogen (blue) and phosphorus (orange) highlighted. (**A**) Side view, matrix is at the bottom. Near the outer membrane’s surface, mutually perpendicular planes of hemes *a* and *a*_3_ are seen, as well as the copper centers (binuclear Cu_A_ and mononuclear Cu_B_, brown spheres). (**B**) View from the outer surface of the membrane. (**C**) Enlarged part of (**B**). Subunit III is located on the right side of the image.

Figure 1 illustrates the similarity in the arrangement of lipid molecules between the type A oxidases. The 3D structures of CcO from mitochondria (green) and *R. sphaeroides* (grey) are overlaid. One can note that the lipid molecules tend to cluster in the same regions of the two enzymes, and that the fatty acid chains are arranged in a similar manner.

In the primary sequences of type B enzymes, the sites of interactions with lipids are preserved to a much lesser extent [64]. Tightly bound alkyl chains are also not detected in the crystal structure of the *ba*_3_ oxidase, with a resolution of 2.3 Å (PDB 1XME) [21]. About 20 lipid molecules can be identified in the high-resolution structure of the *ba*_3_ oxidase (PDB 3S8F) crystallized in lipid medium. They cover about a third of the protein’s surface and are considered annular lipids [22]. The superposition of the structures shows that at least two lipid-binding clusters still spatially coincide in the type A and type B enzymes.

The role of lipids in the structure of the type C heme–copper oxidases has not yet been discussed in detail. It is known that the lipid composition of the membrane significantly affects the amount of the *cbb*_3_ oxidase in *R. capsulatus* cells [65], as well as the interaction of the binuclear center with ligands [66]. Ornithine lipids in the membrane of *R. capsulatus* promote proper folding of the *cbb*_3_ oxidase, and increase its resistance to degradation [65,67].

## 3. Detergents

Taking into account a large number of known terminal oxidases, as well as the wide variety of detergents currently available, we will not pretend to describe their interaction completely. As in the previous section, we consider the most typical and interesting examples.

There is considerable experience regarding the isolation of terminal oxidases using non-denaturing detergents of various chemical structures. The most popular are anionic detergents (bile salts; lauryl sarcosinate), zwittergents (e.g., N-dodecyl-N,N-dimethyl-3-ammonio-l-propane sulfonate, syn. Zwittergent 3-12 or SB-12) and non-ionic detergents (polysorbates, syn. Tween; sorbitans, syn. Emasol, t-Octylphenoxypolyethoxyethanol, syn. Triton X-100; alkyl glycosides).

The original protocol for isolating mitochondrial CcO included the use of cholate or deoxycholate [68,69]. This allowed one to obtain a well-purified multi-subunit enzyme, but with an extremely low activity. Later it was shown that bile acid anions are inhibitors of the cytochrome oxidase activity [70,71]. However, the preparation obtained using (deoxy)cholate could be significantly activated by synthetic non-ionic detergents, such as Emazol or Tween [47]. Thus, Emazol-4130 increased the activity of CcO isolated with cholate by approximately 3.5 times [69]. The enzyme isolated with Emazol instead of cholate remained active during months of storage. The reason for the activating effect of Emazol was associated, in particular, with its beneficial effect on the interaction of CcO with cytochrome *c* [72]. Another possible explanation for the effect of Emazol and Tween on CcO was the putative “fluidity” of the lipid environment, created by the alkyl tails of these two detergents [48]. This reason became irrelevant after the CcO activity in dodecyl maltoside was shown to be an order of magnitude higher than in Tween-20, despite the fact that the alkyl tails of these detergents are the same [73].

To obtain a catalytically active preparation, (deoxy)cholate began to be removed, and replaced with another detergent. However, it was noted that the activity of the enzyme originally isolated with (deoxy)cholate is not fully restored, even in Tween-80 solution [48]. Later, numerous binding sites for bile acid anions with the mitochondrial CcO were discovered. The preparation crystallized using cholate contained 10 molecules of cholate per CcO monomer [74], two to four of which are visible in the 3D structure [11,62]. The structural similarity of cholate and ADP molecules gave researchers a reason to believe that cholate binding marks putative sites of regulatory interactions between CcO and nucleotides [56,74]. However, to date, the existence of only one such site (the so-called “allosteric inhibition” site), located on the matrix surface of subunit IV, can be considered to be proven [54]. In some cases, the inhibitory effect of cholate and deoxycholate on the mitochondrial CcO may be due to a change in the quaternary structure of the enzyme. Indeed, solubilization with nonionic detergents or phospholipids in the presence of an equimolar amount of bile salts, or the zwittergent CHAPS, which is a bile acid derivative, leads to the transition of the eukaryotic CcO into a dimeric form, the activity of which is lower than that of the monomeric one [75]. However, cholate and deoxycholate still inhibit the dimeric form of the enzyme from bovine heart, as well as the CcO from *R. sphaeroides*, which does not form dimers [76]. We believe that in the latter case, the inhibition is due to the binding of the bile anion in the regulatory BABS site, located near the entrance of the K-proton channel (see below). This is confirmed by X-ray structural data, revealing a bound cholate (the so-called “matrix” cholate) in the BABS of the CcO dimer [11], and a bound deoxycholate at a similar location of CcO from *R. sphaeroides* [41].

Despite the above, cholate is still often used in the isolation of CcO, provided that its concentration in the final preparation is strictly limited. Meanwhile, since the late 1990s, the isolation procedure with the use of non-ionic detergents, such as Triton X-100 and dodecyl maltoside, has become popular [77].

Unlike most detergents used to isolate membrane proteins, Triton X-100 consists of a large hydrophobic “head” (4-tert-octylphenol group) and a hydrophilic “tail”. Using Triton X-100, it is possible to isolate a multi-subunit, highly purified, and almost completely inhibited CcO preparation. As in the case of (deoxy)cholate, the inhibition is reversible, and the replacement of the detergent converts the enzyme into its active form. CcO from bovine heart may exist in Triton X-100 solution as dimers or monomers, depending on the conditions. Complete monomerization is caused by high concentrations of Triton X-100 (at least 5 mg per milligram of protein, or 80–800 mM) at pH ≥ 8.5 [78,79,80]. At concentrations of Triton X-100 in the range of 0.1–1% (corresponding to 1.6–16 mM), the enzyme remains almost completely in the dimeric form [48,80].

Recently, the effect of Triton X-100 on CcO from bovine heart has been carefully studied [81]. Submillimolar concentrations of the detergent caused the reversible inhibition of oxidase activity, whereas the partial peroxidase activity of the enzyme remained intact. The inhibition manifested itself, in particular, as the disruption of electron transfer between hemes *a* and *a*_3_. The action of Triton X-100 was prevented and reversed by dodecyl maltoside, apparently due to the competition between two detergents for the site of specific interaction with the enzyme. The described effects can be most comprehensively explained by the specific interaction between the hydrophobic fragment of the Triton X-100 molecule and the BABS regulatory site (see below).

Nonionic detergents from the class of alkyl glycosides are known to be highly effective in the extraction of membrane proteins. Their additional advantage is that, due to the high CMC value, the detergent can be easily replaced by dialysis. The activity of CcO solubilized with alkyl glycosides substantially depends on the nature of both the hydrophilic “head” and the alkyl “tail”. The influence of various alkyl glycosides on the mitochondrial CcO was thoroughly studied [73]. The complete extraction was achieved with both octylglucoside and dodecyl maltolside (syn. lauryl maltoside), while the catalytic activity was an order of magnitude higher with dodecyl maltoside. Notably, a rather high concentration of dodecyl maltoside (0.5%, that is 10 mM) was used in [73]. According to our data, at concentrations above 0.25% this detergent inhibits the oxidase activity by 1.5 times [81].

In a number of cases, alkyl glycosides can be considered to be structural analogues of phospholipids [17]. The effects of dodecyl maltoside on CcO’s activity may reflect the interaction of the enzyme with endogenous lipid molecules in vivo. In the 3D structure of CcO from *R. sphaeroides*, a decyl maltoside molecule is visible, being tightly bound at the boundary of the amphipathic area of BABS. Its alkyl tail lies in a groove near the site that binds the steroid moiety of deoxycholate, and the maltoside head is able, in some positions, to reach the hydrophilic part of the steroid-binding motif [41,42]. Approximately at the same location, in the 3D structure of the mitochondrial CcO, a molecule of phosphatidylethanolamine is tightly bound [41]. According to our estimates, the dissociation constant of the complex of dodecyl maltoside with CcO from bovine heart is 0.5–1.5 mM [76,81,82,83].

**Figure 2:**
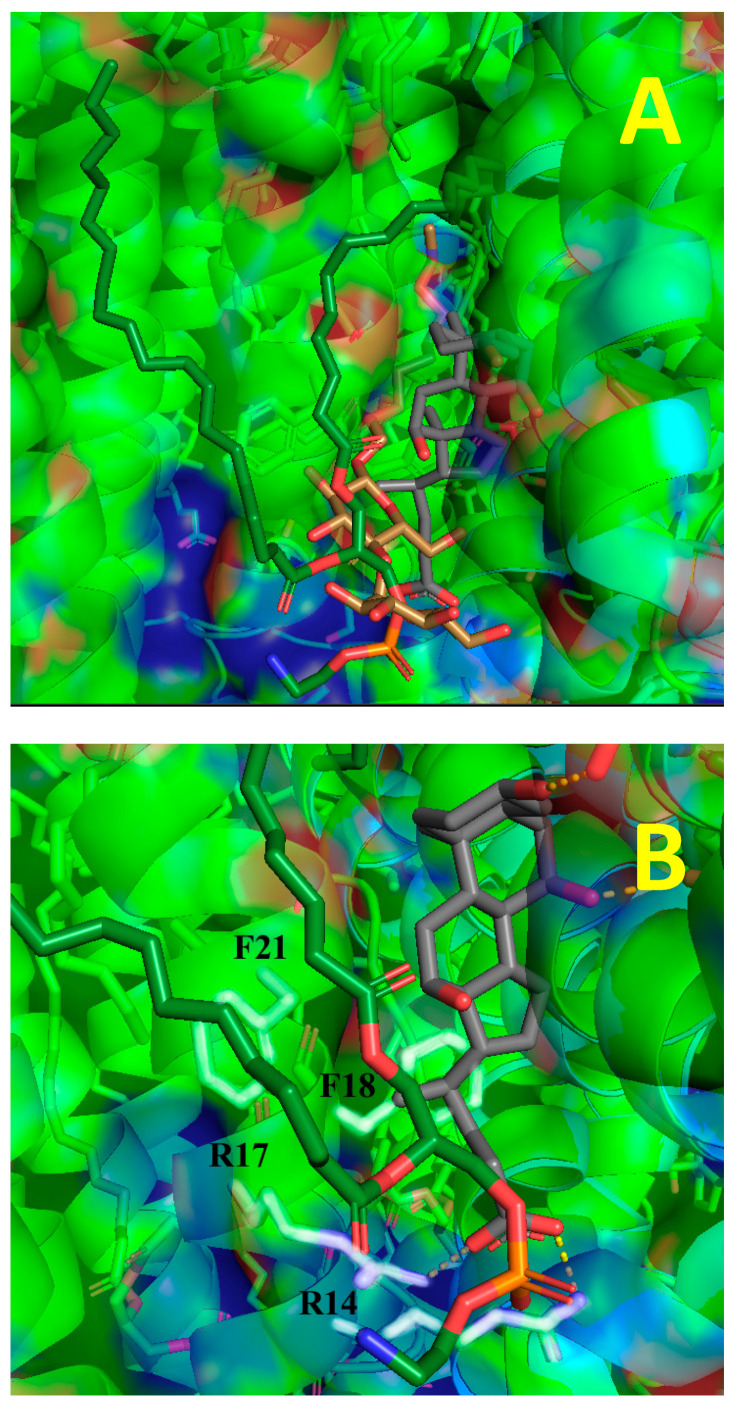
BABS with the ligand and adjacent endogenous lipid. (**A**) The BABS region from the mitochondrial dimeric CcO structure (PDB entry 5B1A) is depicted as a protein surface. The endogenous phosphatidylethanolamine molecule is shown by dark green sticks. Decyl maltoside (brown sticks) from *R. sphaeroides*’s CcO structure (PDB entry 3DTU) is superimposed on the image in the appropriate location. The cholate molecule (grey sticks) is bound in the hydrophobic cavity formed by subunits I, II and VIa (from the opposite monomer). Side view, matrix is at the bottom. Oxygen and nitrogen atoms are colored in red and blue, respectively. (**B**) The same (except for the detergent from the bacterial enzyme structure), but enlarged. The residues of subunit VIa, presumably involved in interactions with cholate (Arg14, Arg17, Phe18 and Phe21), are indicated and tinted in white.

Figure 2 shows the BABS region in CcO from bovine heart mitochondria. At the border of the hydrophobic cavity, which is a binding site for cholate and other steroid-like molecules, endogenous phosphatidylethanolamine is bound. Presumably, in the solubilized preparation it is replaced by a molecule of detergent (dodecyl maltoside). Indeed, a decyl maltoside molecule is revealed in the structure of the bacterial enzyme, at a similar location (see panel A). Panel B demonstrates that the BABS ligands can form bonds with many functional groups of the protein. Indeed, hydroxyls 7 and 3 of cholate are at a distance of 2.8 Å from the O-containing moieties E62_II_ and T63_II_, and the carboxylate moiety of cholate is at the same distance from the nitrogens of Arg17_VIa_ and Arg14_VIa_ (see yellow-dotted lines). This is close enough to form hydrogen bonds. In their turn, Phe18_VIa_ and Phe21_Via_, are likely to form hydrophobic interactions with cholate.

The same types of detergents are used when solubilizing bacterial terminal oxidases, as in the case of the mitochondrial CcO. Some variations may be dictated by differences in the lipid composition of the membranes in different microorganisms. The possibility of performing rapid, one-step isolation of His-tag-bearing bacterial mutant proteins makes the choice of other conditions, including the detergents used, to be somewhat less critical. However, the use of dodecyl maltoside throughout the isolation, or at least at the final stages, is currently considered as the best choice [28,58,84,85].

The isolation of the His-tag-containing quinol oxidase *bo*_3_ from *E. coli* using Ni-NTA affinity chromatography has been described [18]. The membrane solubilization was carried out in three ways: by Triton X-100 in combination with octyl glucoside, by sucrose monolaurate, and by dodecyl maltoside. In the first case, the detergent was then replaced with an anionic N-lauroyl-sarcosine (syn. Sarkosyl), since for the *bo*_3_ oxidase, as for the mitochondrial CcO, Triton X-100 is a strong reversible inhibitor. In all three cases, the resulting preparations were well purified, homogeneous, and catalytically active. It is noteworthy that when using sucrose monolaurate and dodecyl maltoside, the final preparation contained one mole of the bound ubiquinone, while isolation with Triton X-100 resulted in a nearly ubiquinone-free enzyme.

In the classical procedure for the isolation of the quinol oxidase *bd*, the zwittergent SB-12 was used for solubilization, which was replaced with cholate during the purification, and at the final stage with Tween-20 [86]. This procedure yields a homogeneous, well-purified preparation with a sufficiently high catalytic activity, the absolute values of which are obviously determined by the choice of the reducing substrate. However, the properties of the isolated and membrane-bound cytochrome *bd* are not identical, since they show a difference in terms of ligand binding [87]. The classical inhibitor of the oxygen reductase reaction, cyanide, normally binds to the oxidized high-spin heme *d*, mimicking the oxygen molecule. As can be seen from the spectral changes, in the isolated solubilized enzyme, the low-spin heme *b*_558_ also exhibits reactivity with respect to cyanide. The treatment of the membrane preparation with a detergent leads to the same result. Notably, reconstitution of the isolated *bd*-I oxidase into asolectin proteoliposomes restores a ligand-reactivity pattern that is typical of the enzyme in the bacterial membranes. The reduced form of the enzyme is characterized by a reaction with another oxygen mimetic, the CO molecule. In the membrane preparation, this ligand interacts with the binuclear center, causing characteristic spectral changes in heme *d* and, in part, heme *b*_595_. After solubilization, CO noticeably binds to heme *b*_558_, as well. As in the previous case, the reconstitution of the isolated cytochrome *bd*-I into asolectin membranes restores its native CO-binding properties. According to the author’s assumption, the interaction of heme *b_558_* with one of the axial ligands, Met393, is weakened in the solubilized preparation. As a result, this ligand can be replaced by a stronger exogenous ligand, cyanide or CO. The described situation is similar to the well-known phenomenon of cyanide and CO binding to the oxidized and reduced forms of cytochrome *c*, respectively, as a result of weakening the bond of the low-spin heme with axial methionine [88,89]. It is noteworthy that the treatment of the membrane-bound *bd*-I oxidase with different detergents (anionic lauryl sarcosinate, SB12 zwittergent) gives the same effect. Currently, dodecyl maltoside is used in all stages of purification, resulting in a four-subunit (CydA CydB CydH CydX) preparation of the *bd*-type quinol oxidase [28].

## 4. Potential Ligands of the Regulatory Sites

The Bile Acid-Binding Site was described, and then characterized in detail, in a series of studies from Ferguson-Miller’s laboratory [41,42,43,90,91]. The experimental object of this group was CcO from *R. sphaeroides*. They found that the activity of an enzyme mutated at Glu101_II_ (a critical residue of the K proton channel that corresponds to Glu62_II_ of the mitochondrial enzyme) significantly recovers in the presence of low concentrations of cholate or deoxycholate [41]. Furthermore, in the 3D structure of the *R. sphaeroides* enzyme, a tightly bound deoxycholate molecule (used during crystallization) was observed in the hydrophobic cavity, at the junction of subunits I and II, directly at the entrance to the K-channel. The carboxylate group of deoxycholate was located at the border of the channel with the cytoplasm and, apparently, functionally replaced the missing glutamate in facilitating the initial capture of the proton. The cholate molecule was similarly located in the CcO dimer from bovine heart mitochondria. In this case, the hydrophobic cavity was formed, in addition to subunits I and II, by the VIa subunit of the opposite monomer.

As shown in our laboratory, the addition of cholate or deoxycholate to the solubilized dimeric mitochondrial CcO at low concentrations (less than 1.5 mM) induces a concentration-dependent activation of the enzyme up to 1.5-2–fold. With a further increase in the bile salt concentration, this activation is replaced with inhibition [76]. The activation phase can be explained by a local increase in the concentration of protons around the bile acid anion, at the entrance to the K-channel. This interpretation is consistent with the earlier data, regarding the activating effect of exogenous lipophilic anions on *R. sphaeroides* mutants with replacements of the residues located at the mouth of the K or D channels [41,92,93,94]. Presumably, lipophilic anions, in this case, play the role of a proton-harvesting antenna, similar to endogenous carboxylates in bacteriorhodopsin [95]. At higher concentrations, cholate is less likely to dissociate from the binding site and thus inhibits the K channel. As discussed in [76], there are two possibilities for the bound cholate to impair proton transfer through the K-channel. The first is associated with the changes in pK_a_ values of critical residues. Indeed, the hydroxyl-7 of cholate is at a distance of 2.8 Å from the carboxyl of the input glutamate E62_II_, and the hydroxyl-3 is at the same distance from the hydroxyl of the neighboring T63_II_. This is quite sufficient for hydrogen bond formation, and, as a result, the effective pK_a_ value for E62_II_ should increase sharply. The second possible mechanism of the inhibition may be via an indirect influence on the remote residues, resulting in a disruption of the hydrogen bond network within the K-channel. An example of such a remote impact on the K-channel, achieved by blocking its key residue K369 in the “down” conformation, is discussed in [44].

Besides bile acid anions, many other amphipathic molecules, including detergents, fatty acids, and porphyrins, have been shown to influence the CcO activity in the Glu101_II_ mutant from *R. sphaeroides* [42]. Based on the structural and spectral data, the authors postulate that BABS has a specific affinity for amphipathic ligands, the binding of which causes disruption of the proton conductivity of the K-channel, as well as the disruption of electron transfer between hemes *a* and *a*_3_. The study analyzes, in detail, the action of various agents added in different combinations, and concludes that there are two closely spaced and partially overlapping binding sites at the entrance to the K-channel. Later, on the basis of mutagenesis data, this point of view was partly revised [91]. At present, it is considered that most amphipathic ligands bind at a single BABS site, which is a fairly large cavity lined with hydrophobic residues. The transmembrane helices VII and VIII of subunit I and the transmembrane helix II of subunit II border the cavity. Its size is large enough to accommodate even a 3 kDa peptide in α-helical conformation [76].

Currently, the regulatory function of BABS is not in doubt. At the same time, due to the multispecificity of the site, it cannot be ruled out that its true physiological ligand is still unknown. Three computational approaches were used to identify the most likely candidates (ROCS comparison of ligand shape and electrostatics, SimSite3D comparison of ligand binding site features, and SLIDE screening of potential ligands by docking) [43]. The list of the most likely ligands included steroids, nicotinamides, flavins, nucleotides, retinoic acid, and thyroid hormones. As shown in the same study, some of the listed substances actually modulate the activity of the wild-type and mutant CcO from *R. sphaeroides*. The most potent inhibitors were fusidic acid, cholesteryl hemisuccinate, retinoic acid, and T3 thyroid hormone. At a slow enzyme rate, the activity was also sensitive to an ATP analog and GDP.

Although the computer analysis placed steroids among the most likely BABS ligands, their effect on the activity of the bacterial enzyme could not be demonstrated [43]. This was later investigated in our laboratory, using CcO from bovine heart mitochondria [82]. The activity of the solubilized enzyme was completely inhibited by estradiol, testosterone, dehydroepiandrosterone, progesterone, as well as the secosteroides cholecalciferol (vitamin D3) and ergocalciferol (vitamin D2). The true K_i_ value varied from 9 μM (cholicalciferol) to 370 μM (estradiol). The possibility of interaction between estradiol and mitochondrial BABS is supported by the results of docking [83]. The same was done for testosterone, cholecalciferol and cholesterol.

Some characteristic features of steroid inhibition described in [82] indicate that BABS is the site of action for the enzyme. First, the apparent K_i(app)_ value increases linearly with increasing concentrations of dodecyl maltoside, which indicates the competition of the inhibitor with dodecyl maltoside for binding to the enzyme in a ratio of 1:1. This is consistent with the above structural data on the binding of decyl maltoside at the border of bacterial BABS, with the possibility, in some conformations, to overlap with this site [41,42]. Secondly, the partial peroxidase activity of CcO remains insensitive to steroid concentrations that cause the complete inhibition of the oxidase reaction, which is a typical trait for the K-channel mutants. The resistance of the peroxidase activity is explained by the fact that the BABS ligands block the proton K-channel, which is not involved in the peroxidase stage of the catalytic cycle [96,97]. Thirdly, the rapid-mixing data showed that estradiol inhibits the transfer from heme *a* to heme *a*_3_, which is also a characteristic sign of disruption to the K-channel.

The same three peculiarities distinguish the inhibition of the mitochondrial CcO by thyroid hormones T3 and T4 [83]. In this case, the K_i_ value is 100–200 μM and inhibition was observed both in terms of the solubilized and, under certain conditions, the membrane-bound enzyme. In the same study, T3 hormone was docked in mitochondrial BABS. Thyroid hormone T2 at low concentrations had no effect on the oxidase activity, which confirms the specificity of the effect in relation to T3 and T4. At the same time, the partial activity of superoxide generation by the mitochondrial CcO was extremely sensitive to all three thyroid hormones (K_i_ = 0.3–5 μM). We explain this by assuming that the mitochondrial CcO has two sites of interaction with thyroid hormones. The first is BABS, which binds T3 and T4; the second is located on one of the small regulatory subunits of the enzyme, and binds T2, T3 and T4. The hormone bound at the second site prevents the interaction of oxygen with free radical groups that periodically appear on the surface of the operating enzyme. It is very likely that the second site coincides with the T2 binding sitepreviously revealed on subunit Va [98,99]. Therein, T2 binding has been shown to cause small changes in the absorption spectrum of the enzyme and the activation by about 1.5 times. Both effects were explained by the release from the allosteric inhibition induced by ATP.

We also hypothesize that the inhibition of CcO by Triton X-100 may result from a specific interaction with BABS [81]. The effective concentrations of Triton X-100 (K_i_ = 300 μM) are comparable to those of estradiol. Dodecyl maltoside competes with Triton X-100 for binding in a ratio of 1:1. Triton X-100 does not affect the peroxidase partial activity of CcO but suppresses electron transfer between hemes *a* and *a*_3_. Note the earlier study [100] that states for the first time that proton transfer in the K-channel is reversibly inhibited by Triton X-100. We believe that the 4-tert-octylphenol moiety of Triton X-100 spatially mimics the steroid molecule, and thus acquires an affinity for BABS. This is supported by the known property of 4-tert-octylphenol to interact with estrogen receptors (e.g., [101,102]).

It should be noted that the hypothesis regarding the regulation of the mitochondrial CcO by steroid and thyroid hormones is physiologically relevant. As discussed in [82], the expected content of steroid sex hormones in the mitochondrial membrane is in the same concentration range as the K_i_ values obtained in the experiment. In the case of thyroid hormones, the situation is less obvious, since their content in blood plasma is three orders of magnitude lower than that of steroids. However, as discussed in [81], the content of thyroid hormones in tissues is much higher. In addition, there is a wide range of values between individual organs, animal species and physiological states. Taking this into account, the participation of T3 and, especially, T4 in the regulation of CcO through a direct interaction with BABS seems quite plausible.

Besides the relatively low-molecular-weight ligands of BABS discussed above, more recently, we have shown that a 3 kDa peptide can perform the same function [76]. Artificially synthesized P4 peptide is a typical cell-penetrating peptide. It is composed of two flexibly bound modified α-helices from the M1 protein of the influenza virus, each containing a cholesterol-recognizing CRAC motif. It turned out that P4 and some of its analogs specifically and completely inhibit the solubilized and membrane-bound CcO from mitochondria and *R. sphaeroides* (K_i_ = 3 μM). The competition for binding between P4 and dodecyl maltoside in a ratio of 1:1, as well as the competition between P4 and deoxycholate, has been observed. The peroxidase partial reaction was resistant to inhibition. The size estimation shows that the P4 peptide can penetrate BABS partially or even entirely. The comparison of P4 mutant variants showed that the inhibitory effect requires the presence of a Trp residue in the primary sequence. This may be due to the inherent ability of tryptophan to anchor the peptide at the interface between the lipid and aqueous phases [103]. The inhibition itself is most likely caused by the influence of certain amino acids, comprising inhibitory peptides, on the dynamics of Glu62_II_ protonation. These can be, for example, positively charged Lys and Arg residues included in the CRAC motif.

Based on the cryogenic electron microscopy data, another steroid-binding site has been recently described in the monomeric mitochondrial CcO [44]. This “Steroid-Binding Site of a Monomer” (SBSM) lies in the hydrophobic gap between the V, VI, and VII transmembrane helices of subunit I. Although being separated from BABS by about 20 Å, SBSM nevertheless is located in the vicinity of the K-channel entrance. According to the authors, the steroid molecule bound near the VII helix restricts the mobility of the K319 side chain, which leads to disruption of the proton conductivity in the K-channel. In the dimer, SBSM must be closed for interaction with ligands, since it is occupied by the N-terminal portion of the VIa subunit of the opposite monomer. Additionally, the SBSM must be closed in the bacterial CcO, since it is shielded by subunit IV.

**Figure 3:**
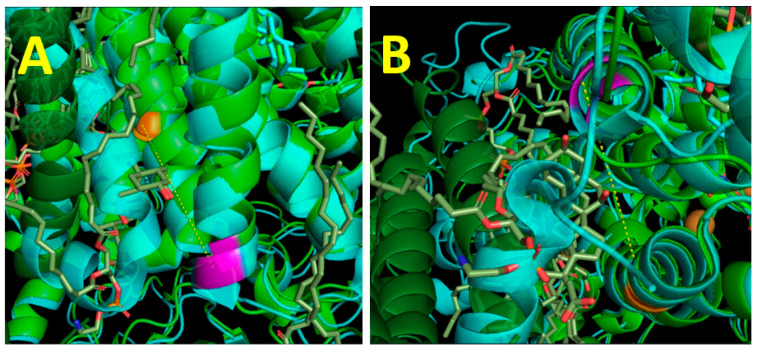
Binding sites for amphipathic ligands in mitochondrial CcO. (**A**) Side view. (**B**) View from the inner membrane surface. The structures of the dimer (formed by light green and a deep green monomers; PDB entry 3ASO) and the monomer (cyan; PDB entry 5Z62) are superimposed. In the center of the image in (**B**), one can see the NDUFA4 subunit, which is present only in the monomeric structure and is involved in the regulation of the monomer–dimer transition [56]. Lipids are grey, with red O and blue N atoms. BABS is marked by E62_II_, colored in purple. SBSM is marked by M278_I_, colored in orange. The distance between E62_II_ and M278_I_ (yellow dashed line) is 20 Å. The cholate bound in BABS is grey with red oxygens. The distance from cholate’ hydroxyl 7 to carboxyl of E62_II_ is 2.8 Å, and the nearest distance from the cholate to M278_I_ is about 8 Å.

Figure 3 shows two binding sites for amphipathic ligands in the structure of the mitochondrial CcO. The BABS and SBSM sites are marked by purple and orange colors, respectively. The distance between them is about 20 Å. Both presumably control the K-channel. The ligand binding with BABS putatively changes the pK_a_ of the input E62_II_, which should result in an impairment of the K-channel’s proton conductivity. Steroid binding with SBSM hypothetically restricts the mobility of the M278 side chain, which indirectly leads to the locking of K319 in the “down” conformation, and subsequently breaks the hydrogen bond integrity in the K-channel.

## 5. Concluding Remarks

Over time, the significance of lipids in the functioning of terminal oxidases has become more and more evident. The role of lipids in vivo is clearly not limited to the environment around the enzyme that is favorable for maintaining its functional structure. In different types of heme–copper oxidases, certain tightly bound lipid molecules are similarly located, and contact conserved lipid-binding protein sites, including those situated deep in the structure. It can be assumed that some of these molecules are functionally close to cofactors and can participate in the catalytic activity. Cardiolipin is involved in the functioning of both heme–copper oxidases and *bd*-type quinol oxidases. Its role is manifested both at the level of the oxidase reaction and at the level of activity regulation, which is associated with changes in the quaternary structure of the enzyme.

The ways in which the properties of terminal oxidases can be affected by detergents are quite diverse, in accordance with the diversity of these agents. Structural or chemical inconsistencies with the natural membrane environment can lead to a change in the properties of the solubilized enzyme, as seen in the example of the bacterial quinol oxidases *bd* and *bo*_3_. Cholate and deoxycholate cause reversible dimerization of the eukaryotic CcO, which is mediated by the participation of small subunits VIa and VIb. This process is probably related to the natural, functionally significant dimerization of the enzyme, which proceeds with the participation of cardiolipin. On the other hand, Triton X-100 can cause the transition of the enzyme into a monomeric form. In addition to these effects, bile salts and Triton X-100 cause the reversible inhibition of the A-type heme–copper oxidases. This can be explained by the interaction of (deoxy)cholate and the 4-tert-octylphenol group of Triton X-100 with the BABS regulatory site. In the mitochondrial-like oxidases, dodecyl maltoside binds at the boundary of BABS, and presumably changes the affinity of this site for ligands. We see here a possible imitation of a natural situation, in which a tightly bound endogenous phospholipid can modulate the affinity of BABS for physiological ligands, thereby participating in the enzyme activity regulation.

The BABS site of the dimeric mitochondrial CcO can bind a variety of physiologically active amphipathic compounds (steroid and thyroid hormones, heme degradation products, nucleotides, and even CPPs). The monomeric CcO probably harbors an alternative site, SBSM, which also binds steroids, at least. It is amazing that wherever the binding site is located, amphipathic ligands affect the CcO activity by the same mechanism, i.e., by disrupting the conductivity of the K-proton channel. CcO quaternary structure transitions cause significant changes in enzymatic activity parameters, including the rate and energy efficiency. The reversible transition of the mitochondrial CcO from monomers to dimers strictly depends on the minor subunits VIa and VIb, which, in turn, are cardiolipin-dependent. On the other hand, it is subunit VIa that is involved in the formation of BABS in the dimeric form. Thus, two regulatory pathways mediated, respectively, by the interaction of CcO with amphipathic ligands, and by the changes in its quaternary structure seem to intersect.

## Data Availability

Not applicable.
